# Complex Population Dynamics in Mussels Arising from Density-Linked Stochasticity

**DOI:** 10.1371/journal.pone.0075700

**Published:** 2013-09-23

**Authors:** J. Timothy Wootton, James D. Forester

**Affiliations:** Department of Ecology and Evolution, The University of Chicago, Chicago, Illinois, United States of America; University of Toronto, Canada

## Abstract

Population fluctuations are generally attributed to the deterministic consequences of strong non-linear interactions among organisms, or the effects of random stochastic environmental variation superimposed upon the deterministic skeleton describing population change. Analysis of the population dynamics of the mussel *Mytilus californianus* taken in 16 plots over 18-years found no evidence that these processes explained observed strong fluctuations. Instead, population fluctuations arose because environmental stochasticity varied with abundance, which we term density-linked stochasticity. This phenomenon arises from biologically relevant mechanisms: recruitment variation and transmission of disturbance among neighboring individuals. Density-linked stochasticity is probably present frequently in populations, as it arises naturally from several general ecological processes, including stage structure variation with density, ontogenetic niche shifts, and local transmission of stochastic perturbations. More thoroughly characterizing and interpreting deviations from the mean behavior of a system will lead to better ecological prediction and improved insight into the important processes affecting populations and ecosystems.

## Introduction

Ecologists have long been fascinated by the fluctuations exhibited by natural populations, and interested in elucidating the mechanisms by which they arise. Early work emphasized stochastic environmental factors that counteracted density-dependent regulatory mechanisms [1]. Subsequently, there has been increased appreciation that density-dependent mechanisms can also act through strong feedback pathways [2]–[7] or time lags [8]–[10] to generate strong fluctuations in population dynamics, including seemingly unpredictable chaotic dynamics. A central question in population ecology is the degree to which each of these processes controls population fluctuations. Answering this question might facilitate prediction of population dynamics for economically important species and potentially guide management practices seeking to minimize outbreaks or population crashes.

Stochastic and density-dependent processes have largely been treated as independent alternative factors both conceptually and in modeling studies [1], [11]. Although past work has considered them as alternatives, more recent work has synthesized the two, modeling populations with stochastic additive noise superimposed on a deterministic skeleton [10], [12]–[16]. Theoretical studies have found that both processes can contribute to shape population dynamics [13], and that when the two are combined, substantial shifts in the characteristics of population dynamics can arise [14], [17]–[20].

An alternative mechanism, which we will refer to as density-linked stochasticity (DLS), has not been considered in most detailed descriptions of population dynamics but might also generate strong non-random patterns of population change [21]. In this case, the impact of varying environmental factors external to the population, such as the myriad components of weather or effects of other species, depends on the density of a population of interest. For example, impacts of fire on forest trees might depend on density, as it is easier for fire to spread from one tree to another at higher density [22]; pathogen transmission may behave similarly [23]. Generalist predators, when their abundance is not strongly controlled by prey abundance, might act as a stochastic factor on prey that may vary with prey density due to an aggregative response [21]. When populations have intrinsic structure, some life stages might be more susceptible to fluctuating environmental factors than others. When the composition of these stages varies regularly with population size, stochastic variation will also vary with population size.

Here we present a detailed analysis of the population dynamics of the mussel *Mytilus californianus* that probes the roles of deterministic non-linear dynamics, additive effects of environmental stochasticity, and density-linked stochasticity. We find that the former two cannot generate the strong dynamical patterns that we observed in our data, but that DLS can. In light of this result, we then explore the mechanism of this phenomenon and consider its general implications.

## Study Site and Methods

### Ethics Statement

All necessary permits were obtained for the described field studies. Specifically, all research was conducted from Makah tribal lands, for which we received the written permission required for access to Tatoosh Island by the Makah Tribal Council.

### Data Collection

This study took place in the middle zone of the rocky intertidal community of Tatoosh Island, Washington, USA (48°23′N 124°44′W). In this zone, the community is dominated by the California mussel, *M. californianus*, with a suite of mobile and sessile algal and animal species that occupy interstices between individual mussels or gaps of open space between groups of mussels [24], [25]. As the dominant species in this community, mussels strongly alter the population dynamics of associated species [26]–[29], hence understanding the patterns and mechanisms driving mussel population fluctuations is important for understanding this system. The qualitative pattern of *M. californianus* abundance through time raises the possibility that interesting non-linear dynamics might be operating: populations go through a cycle of population build up followed by sharp declines on a seemingly regular basis. Yet a central factor thought to determine local mussel populations is physical disturbance by large waves [24], a stochastic external event. To better understand the basis for the dynamics observed in this community, we explored the role of deterministic non-linear dynamics and stochastic impacts on mussel populations.

To explore the community dynamics of this system, we began in 1993 to collect annual data on the early-summer abundance of all species contained within replicate 60×60 cm permanent plots. Plots were located on wave-exposed rock benches at four sites scattered around the island in areas dominated by mussels. Fourteen plots were established in 1993, and another two were added in 1994. One plot could not be located in 1994, which generated a gap within its time series. Data from the plots continue to be collected; the data reported here span 1993 through 2010, yielding 17–18 year time series. To provide maximal information on plot dynamics over a range of densities and to allow permanent plot marking with minimal disruption to the system, we initially chose plots in spots that tended to have relatively low populations of mussels. Note that because this study was carried out in the field and we made no intervention to the natural dynamics of the system, the dynamics are not experiencing any transient phase as do most modeling and laboratory studies that use arbitrary starting conditions. For ten quadrats, mussel abundance was quantified as the proportion of plot area covered, which was aided by using a quadrat subdivided into 121 squares. The sampling involved counting squares exhibiting complete cover of *M. californianus*, and integrating mussel cover on an approximately quarter-square basis for sample squares with incomplete cover. A reasonable discrete approximation of these data, which is useful for the analyses that follow, is the presence/absence of *M. californianus* in each of 484 quarter-squares. For the other six quadrats, sampling involved recording the presence of a mussel under 100 fixed points created at the corners of each grid square [30], [31]. Measurement error, which we assessed from repeated sampling of the same plots, was low (0.48%), so uncertainty in parameter estimates is attributed to process error.

### Analysis and Modeling

We analyzed the time series by exploring relationships between mussel abundance at a census point as a function of mussel abundance one or more census points in the past, combined across all plots, using non-linear regression with maximum likelihood, and used simulations of the resulting relationships to determine how well they recreated the dynamical patterns observed in the data. To match the structure of the data (bounded above and below), we used the beta-binomial distribution to model error (see [32], [33] and Text S1 for more details).

To explore patterns of mussel dynamics, we first probed the time series for the order of density dependence (ODD; dependence on various time-lagged densities) using a non-parametric method [10], [34] that uses cross-validation to assess how well a portion of a time series projects the rest of the data given a specified order of density dependence. We carried out this analysis for the time series of each plot, and standardized the results by setting the lowest cross-validation error of each plot to 0. We then averaged the cross-validation index for first through fifth-order density dependence across all plots (a lower average index indicates greater model support).

To explore the role of deterministic dynamics in generating mussel population patterns, we used maximum likelihood methods to fit a fourth-order model of the form:
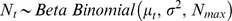


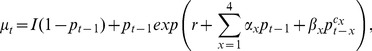
(1)where *N_t_* is the population size at time *t*, *BetaBinomial* is the beta-binomial distribution (see Text S1), *µ_t_* is the mean (deterministic) component of the relationship, *p_t_* = *N_t_/N_max_*, *I* is a term describing immigration from outside the study plots into space unoccupied by mussels, *r* is a density independent rate of growth, *α_x_* describes the effect of linear density dependence at time *t-x*, *β_x_* determines the strength on non-linear density dependence at time *t-x*, *c_x_* controls the strength of non-linearity in density-dependence at time *t-x*, *N_max_* is the number of squares sampled for presence/absence of mussels (484 or 100, depending on plot), and *σ^2^* is the variance around the deterministic component.

The beta-binomial distribution is controlled by two positive-valued shape parameters (*a, b*), which define the beta distribution component, and the sample size (*N*), which defines the subsequent binomial component of the distribution. The mean and variance of the beta portion of the distribution are related to the shape parameters (Text S1). However, because the variance of the beta distribution is not independent of its mean, an overdispersion parameter (i.e., the sum of the shape parameters) can be used to describe spread (Morris 1997). Hence, we fit models of beta-binomial distributions given an expected value (e.g., a function describing predicted mean population size) and rules for the behavior of the variance of those distributions, based on a function describing predicted overdispersion (Text S1).

Equation (1) is a modification of the standard Ricker form of discrete-time population dynamics [16], [35], which has the desirable features that population size can never be negative and that complex dynamics, including cycles and chaos, can be generated. This equation was based on prior mechanistic understanding of mussel populations, which are open to immigration from dispersing larvae in the water column [36]–[38] and dislodged mussels from other parts of the shore [24], [31], subject to strong competition for space [26]–[29] and characterized by local increases in abundance arising from individual growth [24]. The model was also modified by including non-linear and higher-order terms as suggested by the ODD determination, and by other models of sessile marine invertebrates [39]. In light of the literature linking strongly fluctuating dynamics to time-lagged variables [8], [10], [12], and the results of the ODD analysis that suggested fourth-order models might be plausible (although first order models were deemed more likely), we examined 4th order models to ensure that we did not miss possible effects of time lags. As the mechanisms that might lead to higher-order dependency are uncertain, and therefore specific functional forms to use are unclear a priori, we adopted an approach that included general exponential terms for different time lags (e.g., [40]). We also explored simpler versions that included only a one-year lag (time-dependent subscripts of parameters dropped):
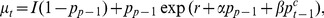
(2)


These versions were applied to the same data series (i.e., capable of exploring up to fourth-order terms), and were evaluated for relative support using AIC analysis [41]. When first-order models were better supported than fourth-order models, we subsequently used the entire data series to estimate a best-fitting model.

We did not explore an alternative model of sessile species population dynamics [39] because it requires accelerating growth to generate unstable dynamics, but *M. californianus* exhibits decelerating growth with age [25, A. Kandur unpubl. data]. As a two-stage model, it also implies second-order lagged dynamics, which were not supported by the ODD analysis. We did, however, explore an alternative, commonly used model [42] that can exhibit a range of dynamic behavior. As with the Ricker model, we modified this model to allow outside immigration and extra non-linearity in the relationship:
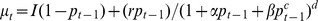
(3)


Because this model generated nearly identical results to Equation (2) (Fig. S1), we do not discuss it further.

Alternative mechanisms for generating strong population fluctuations differ in how stochastic variation is conceptualized. Therefore we compared models that incorporated stochasticity in different ways. First, we followed standard approaches in which stochasticity was modeled as a constant feature in Equations 1–3 (*σ^2^* constant). Second, because we suspected that DLS might be occurring, we also probed models in which stochasticity was a function of density. There are at least two ways to envision stochasticity varying with density. Stochasticity might vary continuously with density if, for example different stages in the population vary somewhat predictably with density, and are affected by different stochastic forces of different strength. Hence, we modeled variance with the following equation, which can take on multiple patterns of variance with abundance and also facilitates efficient parameter estimation in the face of model variance constraints (see Text S1 for further information):
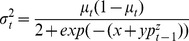
(4)


Stochasticity might also be injected by a process that could vary with abundance, and that creates substantially different dynamics from baseline conditions (i.e. a disturbance). This case can be modeled by a separate set of equations describing dynamics under disturbance conditions [20], with a function describing how the system shifts from undisturbed to disturbed states (a mixture function):
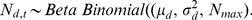
(5)


Here, disturbed conditions are characterized by a mean post-disturbance proportional cover *(µ_d_*) and an associated error *(σ^2^_d_*), assumed to follow a beta-binomial distribution that was constrained to be unimodal. Because we suspected that both effects on stochasticity might be occurring, we incorporated both in our modeling framework. We combined the likelihoods (*L*) of the equations describing disturbed and undisturbed populations, given our data, using a mixture model to describe disturbance probability as a function of mussel abundance:
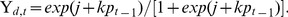
(6)


Hence the log-likelihood (*LL*) equation for this model followed the form:

*LL* = log((1−Ψ*_d_*)*L*+Ψ*_d_L_d_*).
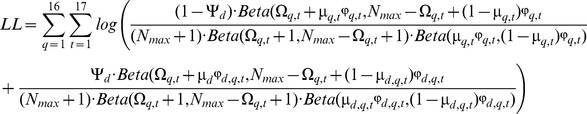
(7)where Ω*_q,t_* is the observed population size in quadrat *q* at time *t*, and *φ* is the sum of the two shape parameters describing a beta distribution, which is functionally related to *μ* and *σ^2^* (see Figs. S2, S3).

We compared different model versions using model selection methods (AIC; [41]), and considered models well supported if their AIC differed from the model with the highest support (lowest AIC) by less than 6 and if a simpler nested model did not have a lower AIC [43]. We also carried out simulations of different model variants. We compared their dynamic characters to those of the data by applying spectral analysis [44], [45] using the fast Fourier analysis function in MATLAB (v. 7.5, MathWorks, Natik, Massachusetts). To mimic the data as closely as possible in this analysis, we carried out the same number of simulations as we had data series (16) starting from the initial conditions in each the different plots, and iterated the models over the same time span as the data (18 yrs). We also visually compared observed population change over an annual time step to 95% confidence intervals expected under different stochastic models by iterating each model 10^6^ times for each 0.002 increment in starting density and plotting *N_t_* against *N_t−1_*.

We checked the robustness of our best-fit models in two ways. First, we compared our best-fitting first-order mean model with constant variance to results from non-parametric curve fitting (distance-weighted least squares) to determine whether the functional shape deviated substantially, which would indicate that our model choice or fitting routine was poor. We also compared a version of the model using a modification of a Hassell-type density-dependent model as described above (Equation 3).

Second, we also checked for robustness of our best-fitting density-linked stochastic model using model averaging, in which parameter estimates for each model considered were combined using the Akaike weights of each model as a weighting factor, based on all model variants considered for either first or fourth order datasets.

## Results

### General Patterns of Population Dynamics

**Figure 1:**
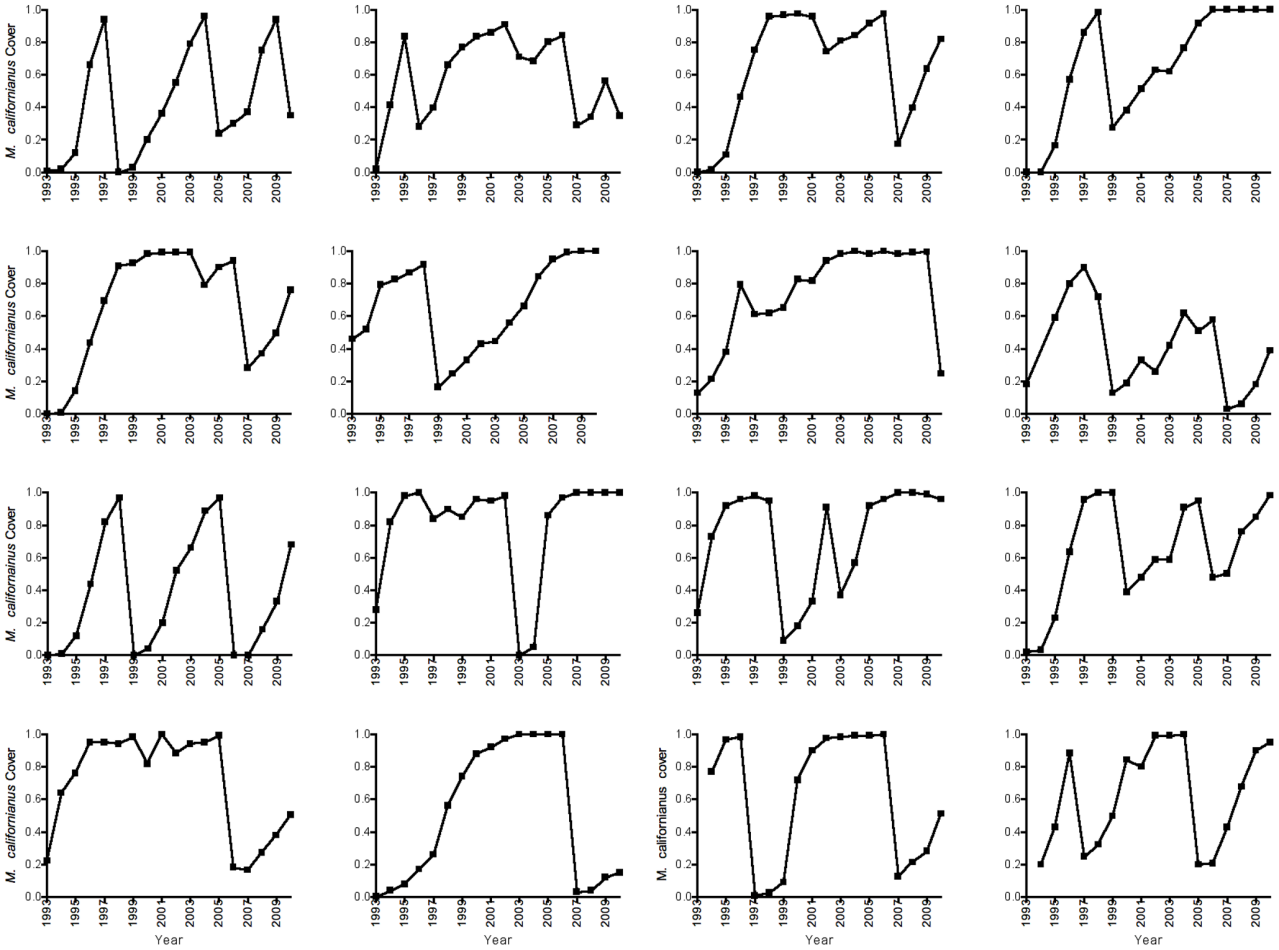
Temporal trajectories of replicated *Mytilus californianus* populations, Data from 16 repeatedly censused 60×60 cm plots on wave-exposed rock benches of Tatoosh Island, Washington, USA over a 18-year period.

Plots of mussel abundance over time follow prior qualitative impressions of temporal dynamics (Fig. 1): mussels generally increase steadily from low abundance, then drop sharply from high abundance. Such strong and regular changes are hallmarks of unstable population dynamics, and suggest underlying deterministic processes such as non-linear over compensatory density dependence or time-lagged density-dependent feedback [2], [5], [46], [47]. Also, the observed fluctuations were not synchronized across plots (Fig. 1), in contrast to the pattern expected if they arose from large-scale environmental forcing.

### Testing Deterministic Non-linear Dynamics

**Figure 2:**
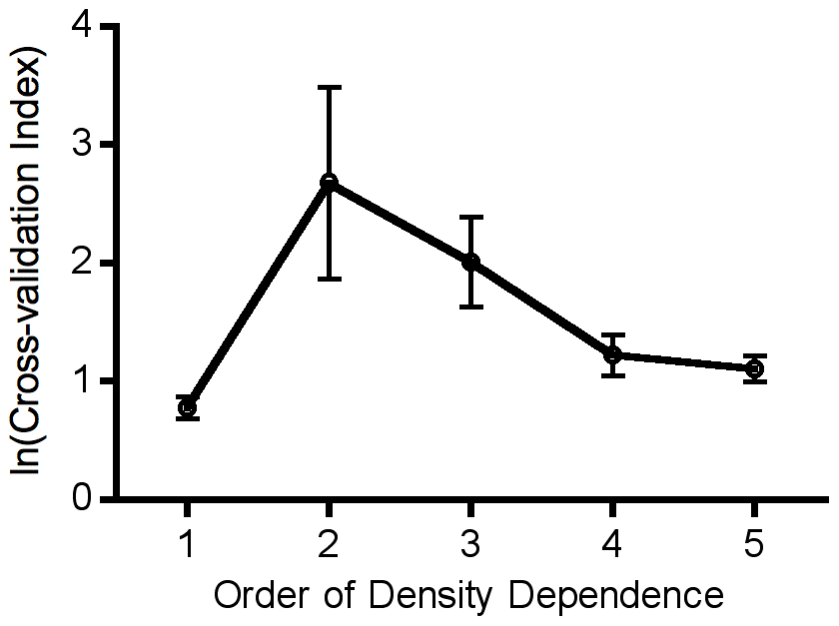
Relative cross-validation scores for models of different orders (lags) of density dependence applied to the 16 empirical time series. Analysis based on a nonparametric test for the order of density dependence [34]. Lower cross-validation scores indicate higher support.

**Figure 3:**
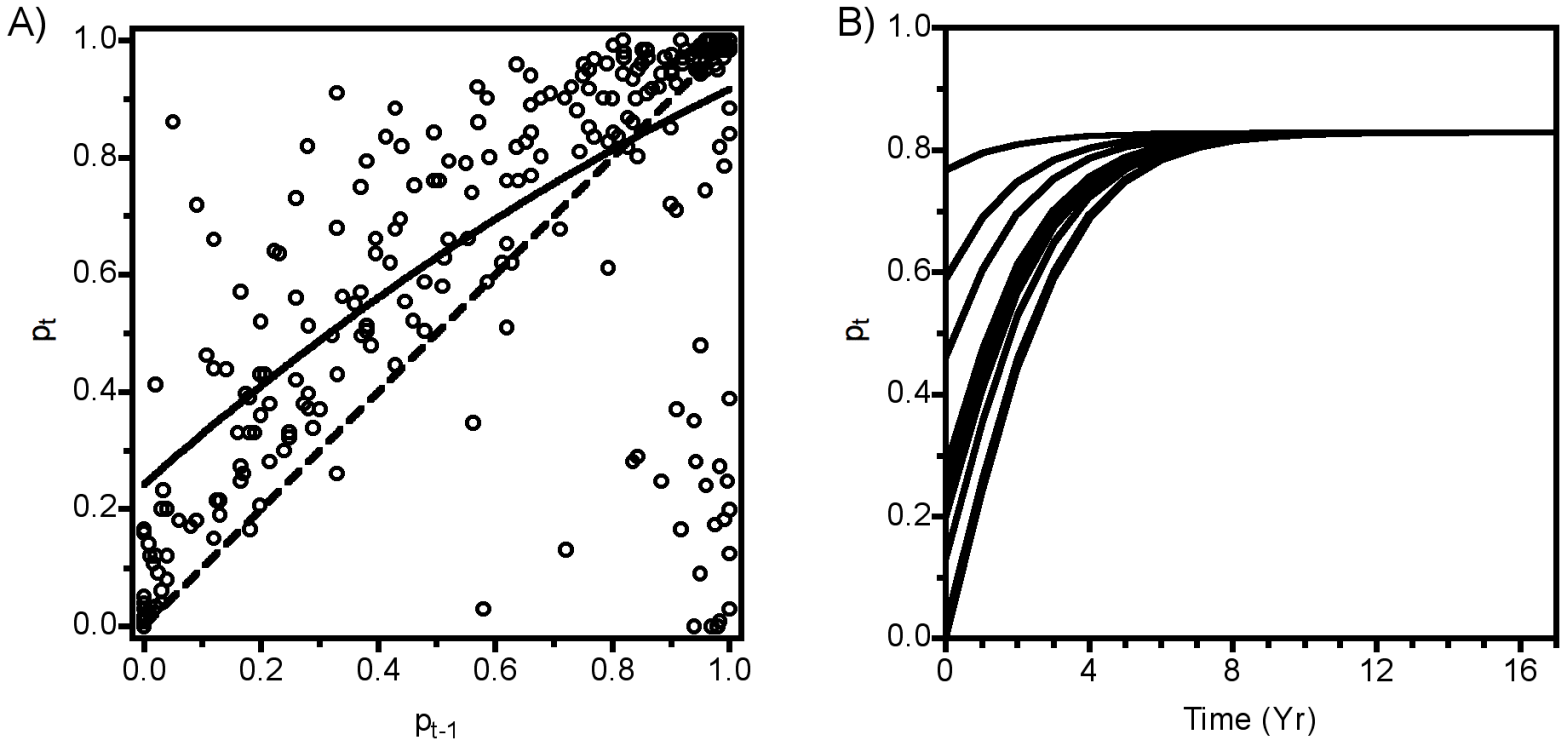
Analysis of deterministic dynamics under constant variance assumption. A) Ricker diagram plotting *Mytilus californianus* population size as proportion of quadrat covered (*p*) at year *t* versus population size in the previous year, with best fitting relationship (solid curve) and steady state line (dashed). B) Deterministic dynamics of best-fitting model to population data. Simulated trajectories start from each of the initial conditions in the empirical plots.

The results of an order of density-dependence analysis (Fig. 2) suggested two possible scenarios: a first-order relationship considered in most classical analyses of population dynamics [2], [16], [35] was most strongly supported, but a fourth-order relationship also performed fairly well. Hence we fit our mussel data to first and fourth-order population dynamics models. The most parsimonious, best fitting model (lowest AIC) was a first-order model with linear coefficients (Table 1; Fig. 3A). The non-linear model also received slightly less support than the linear model when the full, first-order dataset was used (Table 1).

**Table 1:** Maximum likelihood estimates of parameters for constant-variance models of mussel dynamics.

	4-Year Lag Data			1 Year Lag Data	
	Full Model	1^st^-Order Non-Linear	1^st^-Order Linear	Non-Linear	Linear
I	0.234	0.248	0.250	0.239	0.242
r	0.121	0.100	0.040	0.255	0.122
α_1_	−0.116	−0.207	−0.130	−0.408	−0.209
β_1_	0.011	0.019	0*	0.070	0*
c_1_	18.89	29.369	1*	8.032	1*
α_2_	−0.038	0*	0*	0*	0*
β_2_	0.005	0*	0*	0*	0*
c_2_	0.176	1*	1*	1*	1*
α_3_	−0.021	0*	0*	0*	0*
β_3_	−0.010	0*	0*	0*	0*
c_3_	0.351	1*	1*	1*	1*
α_4_	−0.004	0*	0*	0*	0*
β_4_	−0.003	0*	0*	0*	0*
c_4_	0.536	1*	1*	1*	1*
σ^2^ *_est_*	0.034	0.037	0.037	0.035	0.036
-LL	1040.46	1042.74	1043.25	1286.03	1287.50
k	15	6	4	6	4
AIC	2110.92	2097.49	2094.49	2584.05	2583.01
ΔAIC	16.437	2.993	0	1.045	0
w_i_	0.0002	0.183	0.817	0.372	0.628
ER	3709.182	4.467	1	1.686	1

* parameter fixed a priori.

*–LL: Negative log likelihood of model.*

*k: Number of estimated parameters.*

*σ^2^_est_*: Maximum likelihood estimated variance around the mean model.

*AIC*: Akaike Information Criterion value. The best supporting value has the lowest AIC.

*ΔAIC*: Difference between AIC of a candidate model and the best-supported model overall.

*w_i_*: The Akaike Weight of a model.

*ER*: The Evidence Ratio comparing the weight of the lowest AIC model to the weights of other models.

Our model projections (Fig. 3B) provided no evidence that the observed strong population fluctuations could be explained by the deterministic components of the model by themselves: the deterministic skeleton with empirically-derived parameters predicted that the system should be strongly stable, with 82.9% of each plot consistently covered by mussels in the best-fitting model. The predicted dynamics of the non-linear first-order model and full model also exhibited highly stable dynamics. Furthermore, the shapes of non-parametric LOESS fits or a Hassell-type model (Fig. S1) also predicted stable dynamics. In contrast, observed population dynamics regularly exhibited complete dominance by *M. californianus*, and strong fluctuations (Fig. 1). Therefore the strong fluctuating patterns exhibited by mussel populations did not appear to arise from deterministic non-linear or time-lagged dynamics alone.

### Constant Stochasticity Model

**Figure 4:**
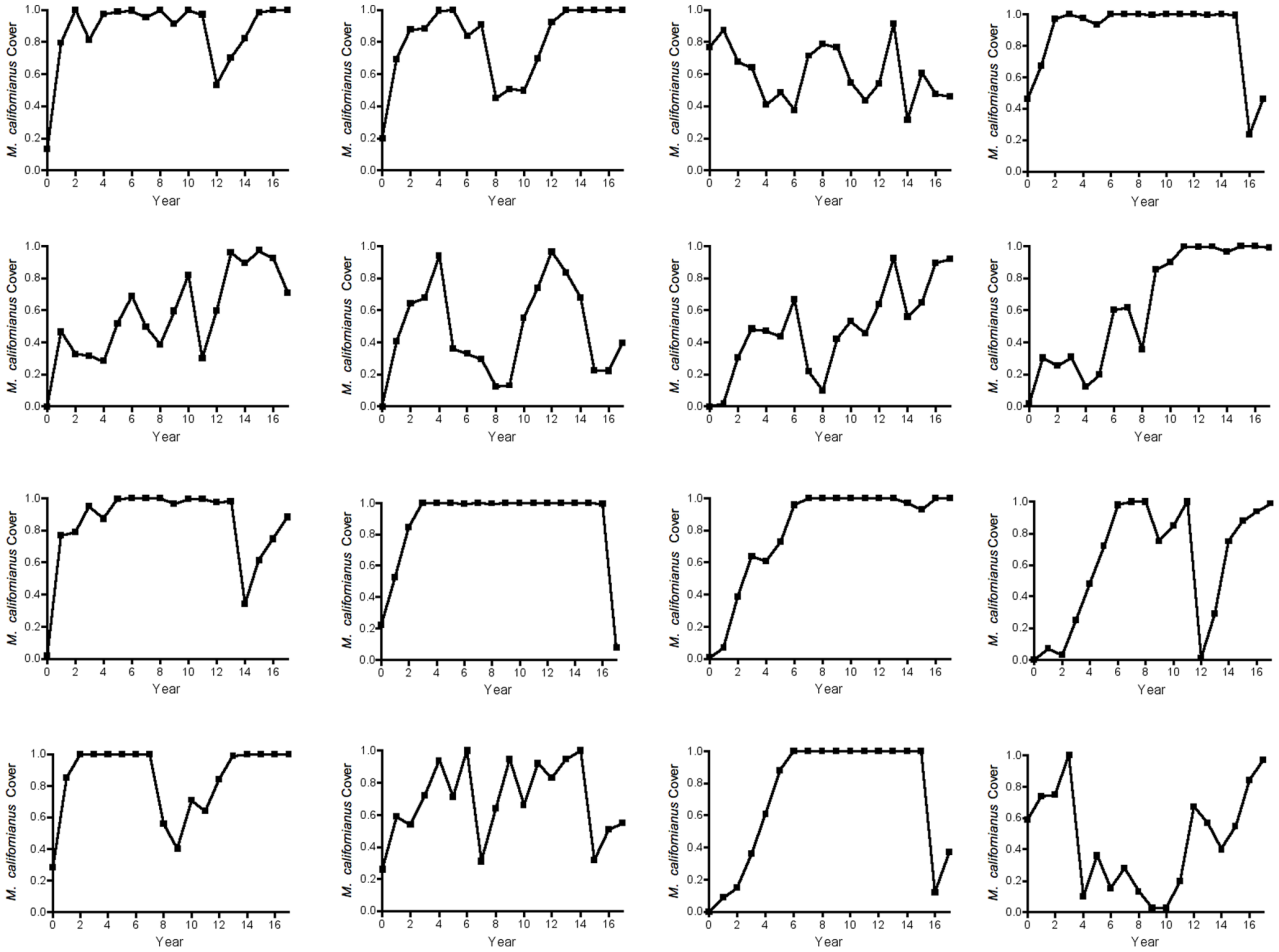
Graphs of 16 simulations of *Mytilus californianus* population dynamics. Simulations used the best-fitting deterministic model skeleton with an added constant stochastic term (Equation 2, Table 1).

**Figure 5:**
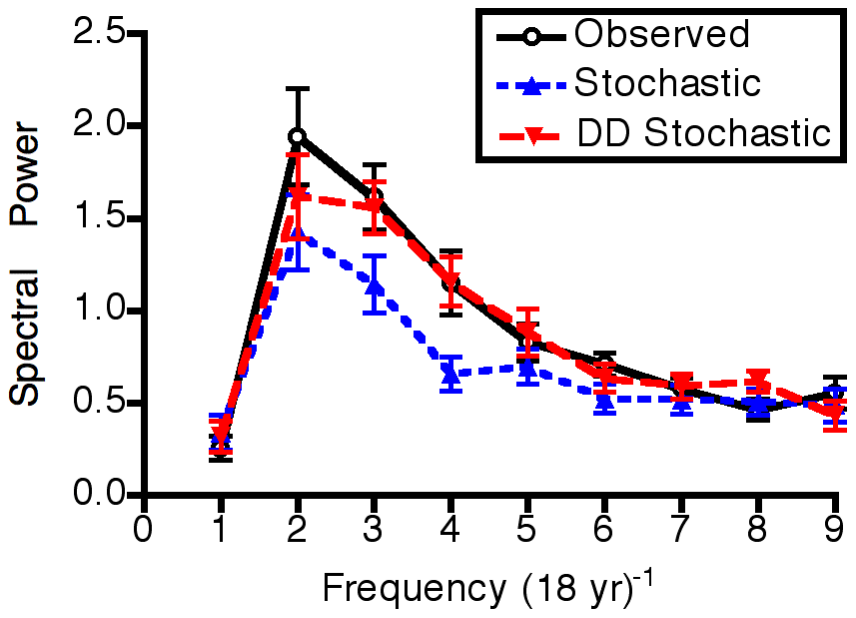
Spectral analysis of observed data (Fig. 1) and of model results both with (Fig. 4) and without (Fig. 7) density-linked stochasticity. The graph depicts the relative contribution of trigonometric functions of different recurrence frequencies spanning the 17-year observation period. Error bars represent one standard error.

**Figure 6:**
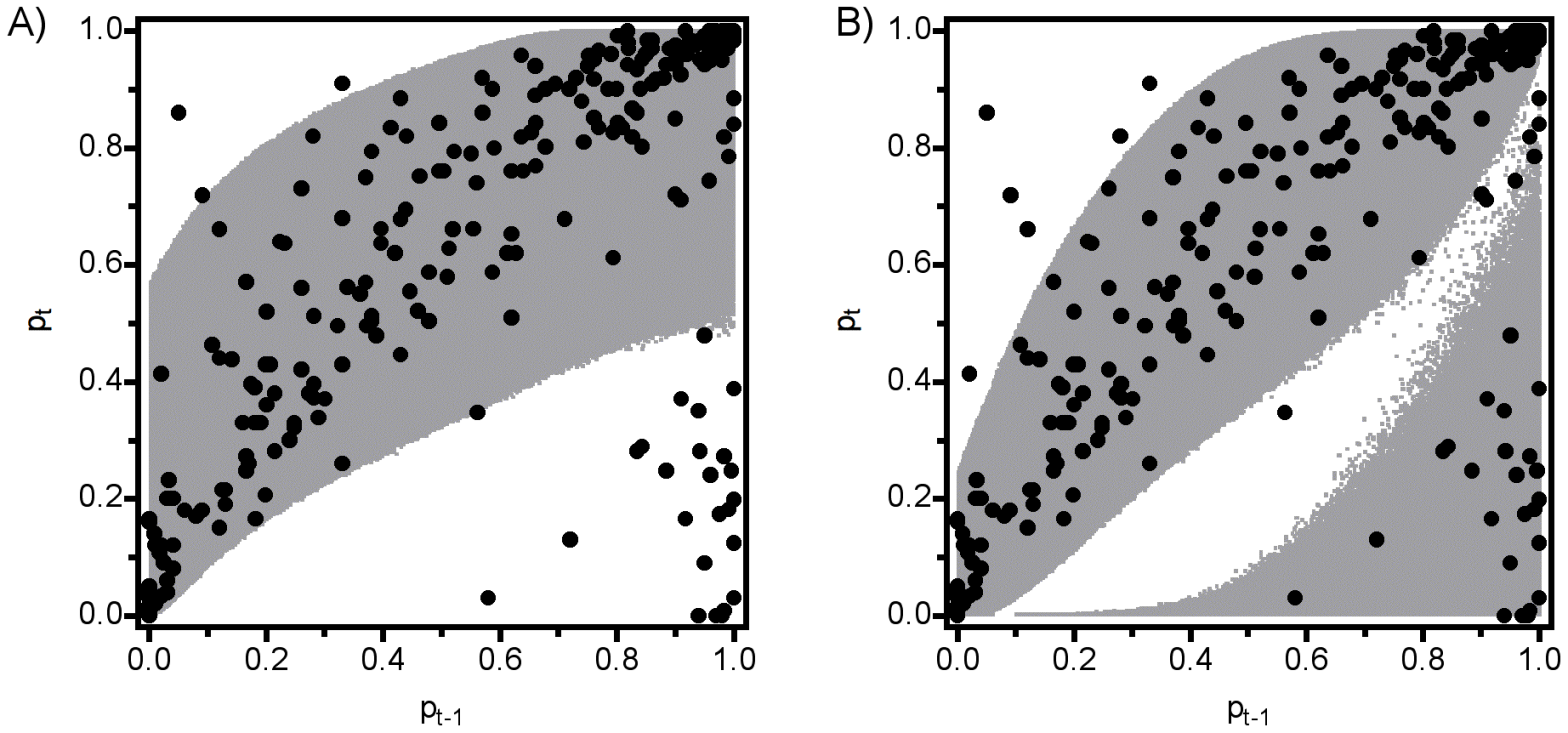
Observed distribution of *M. californianus* abundance at time *t* as a function of abundance the previous year (black points), overlaid on the approximate 95% prediction interval (gray points) derived from one million iterations for each 0.002 increment. A) a standard model with uniform stochasticity, B) a model with density-linked stochasticity. Because the probability distribution for density-linked stochasticity is multimodal, confidence envelopes were derived by summing bins from highest to lower probability until the sum exceeded 95%. Note the large number of observed points outside the 95% contour (lower right) and the lack of observed points in large areas inside the 95% contour for the uniform model.

As would be expected, a model that included constant stochasticity predicted fluctuations in population size through time around an attractor rather than convergence to a stable value as seen in purely deterministic models (Fig. 4). The magnitude of the fluctuations around the attractor, however, was relatively small compared to the fluctuations observed in the actual time series. Spectral analysis of the observed data revealed generally higher temporal variability (high average spectral power), and long-period (low frequency) variation in the data (Fig. 5). Although they also exhibited a relatively dominant low-frequency component, the data from the simulations of the best-fitting stochastic model failed to exhibit the strong variability (i.e., high magnitude of spectral power) of the observed data. Hence the standard approach of adding constant random fluctuations to a deterministic skeleton did not capture the observed dynamics. The comparison of annual population change with confidence intervals generated by the constant stochasticity model revealed systematic inconsistencies (Fig. 6A). Specifically, population growth rates exhibited moderately high variability at low abundance (<10% cover), very high variability at high abundance (>80% cover), and relatively low variability at intermediate abundance.

### Density-Linked Stochastic Model

The model with DLS fit the data much better than one with a constant stochastic term (Table 2, ΔAIC = 147.4). The parameters of the best supported model indicated: a) an increasing disturbance probability with abundance, as expected in prior disturbance scenarios [30], b) a declining undisturbed variance with abundance, perhaps because of a diminishing role of recruitment processes, and c) a positive non linear effect on mean population growth at very high abundance, indicative perhaps of recruitment facilitation by adults [26] or mutual support arising from high interconnection of byssal threads. An alternative model with linear coefficients was also plausible under the model selection criteria of Richards et al. [43] (ΔAIC<6). Further evaluation of potential positive density dependence at high abundance is merited. Parameters generated by model averaging of all model forms considered (Table S1) were similar to those generated by the best-fitting model (Table 2), indicating that these estimates were robust. Confidence intervals for parameters involving lags >1 year encompass null expectations (Table S1), supporting our focus on first-order models. The best-fitting first-order model using all available data had parameter estimates that generally fell within 3% of the parameters of the averaged model (Table S1). The first-order non-linear terms, which are 29% lower than the best estimate, were an exception, but the confidence intervals were well away from null expectations and this term had little effect on model behavior.

**Table 2:** Maximum likelihood estimates of parameters for linear and nonlinear models of mussel abundance dynamics with density-linked stochastic terms.

	4 year lag data			1 year lag data	
	Full Model	1^st^ Order Non-Linear	1^st^ Order Linear	Non-Linear	Linear
I	0.048	0.050	0.053	0.071	0.064
r	0.562	0.546	0.522	0.600	0.593
α_1_	−0.539	−0.552	−0.524	−0.615	−0.596
β_1_	0.007	0.005	0*	0.014	0*
c_1_	38.625	131.266	1*	28.620	1*
α_2_	−0.010	0*	0*	0*	0*
β_2_	0.458	0*	0*	0*	0*
c_2_	5.939	1*	1*	1*	1*
α_3_	−0.006	0*	0*	0*	0*
β_3_	0.919	0*	0*	0*	0*
c_3_	0.030	1*	1*	1*	1*
α_4_	0.006	0*	0*	0*	0*
β_4_	−0.0002	0*	0*	0*	0*
c_4_	2.944	1*	1*	1*	1*
*x*	−3.053	−2.968	−2.959	−2.570	−2.862
*y*	−1.721	−1.929	−2.023	−2.308	−2.222
*z*	11.471	13.701	13.133	8.983	8.434
*µ_d_*	0.399	0.394	0.393	0.392	0.390
*σ^2^_d_*	0.090	0.090	0.090	0.090	0.090
*j*	−3.069	−3.115	−3.143	−3.172	−2.920
*k*	2.155	2.191	2.225	2.288	2.040
–LL	962.36	966.33	967.65	1205.80	1209.03
k	22	12	10	12	10
AIC	1966.73	1956.66	1955.31	2435.60	2438.06
ΔAIC	11.421	1.356	0	0	2.458
w_i_	0.002	0.337	0.663	0.774	0.226
ER	302.08	1.97	1	1	3.42

* parameter fixed a priori.

*w_i_* and *ER* include constant variance versions of the models (Table 1).

**Figure 7:**
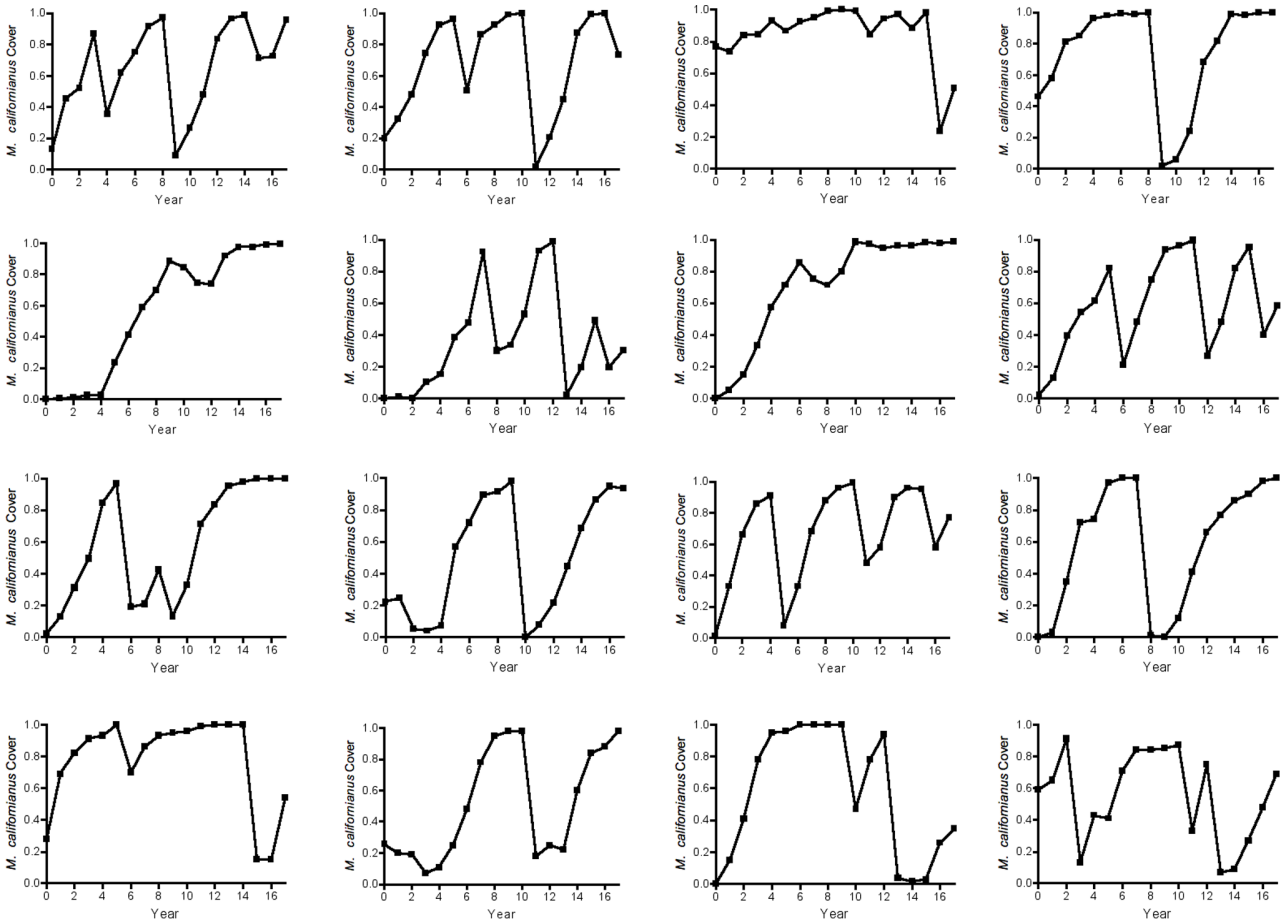
Graphs of 16 simulations of *Mytilus californianus* population dynamics. Simulations used the best-fitting density-linked stochastic model (Equations 3, Table 2).

Simulated data from the DLS model (Figs. 6B, 7) showed a pattern similar to the observed data (Fig. 1), suggesting that DLS was a key process in generating the strong quasi-periodic fluctuations we observed. This conclusion was confirmed by spectral analysis, in which the model with DLS effectively captured the long-period fluctuations observed in the data (Fig. 5). A similar pattern of DLS was generated in output from a model with locally transmitted disturbance compared to a non-spatial mean field model (Fig. S4; Text S2).

## Discussion

Our analysis of mussel population dynamics demonstrated a phenomenon, density-linked stochasticity, which is capable of generating strong, somewhat periodic fluctuations in populations through time. In this case, the seemingly predictable pattern of fluctuations arises because stochastic forces such as wave disturbance have little effect at moderate abundances, leading to predictable recovery times, but very strong effects at high abundance. This situation leads to refractory periods with characteristic recovery times followed by strong population declines at less predictable intervals.

The pattern of DLS exhibited by mussels suggests the important influence of several biologically meaningful processes that need to be accounted for. First, high variability at high abundance is likely to be associated with wave disturbance events, which play an important role in structuring this community [24]. Mussels attach themselves via byssal threads to hard substrates, including neighboring mussels. If a large wave dislodges a mussel, that mussel pulls on any neighboring mussels to which it is attached, leading in many cases to their dislodgement too. This transmission of disturbance from one individual to another is more likely at high abundance, when mussels are more likely to be crowded together and to attach to each other rather than the rock [30]. Hence a stochastic force, wave disturbance, is more likely to act on the population at high rather than low abundance (Text S2).

Second, the relatively high stochasticity at low density is associated with conditions where outside immigration dominates per-capita population growth. Recruitment of mussel larvae from the plankton is responsible for immigration at low densities. However, planktonic larval recruitment is thought to be highly variable because of differences in transport, annual conditions within the water column, and the abundance of other competitor and facilitator species that occupy potential settlement sites [36]–[38], [48].

The DLS observed in the mussel time series has several implications for determining population dynamical patterns. First, because the stochastic component of population change depends on abundance, simply adding stochastic terms to population models is unlikely to capture their essential dynamic character. Second, standard regression methods generally assume that deviations from a fitted function are derived from a distribution that is invariant across the range of the dependent variable (i.e., homoscedastic residuals). Clearly, this assumption is violated when stochastic effects are associated with density. Hence, alternative theoretical and statistical frameworks are required, as outlined in Equations 3–6.

Despite the match in density-linked stochastic model dynamics to observed dynamics, close inspection (Fig. 6B) shows several outliers at low density, which might reflect inadequate characterization of recruitment. These outliers occur in different years, which is inconsistent with large-scale shifts in ocean conditions creating recruitment anomalies. Instead, the abundances of the mussel *M. trossulus* and of filamentous red algae, two taxa that facilitate recruitment of *M. californianus* [49], are positively associated with outliers and a model including their effects has a significantly better fit (ΔAIC = 13.8). Including barnacles, which facilitate recruitment in other mussel species [50], provided no substantial improvement in fit, in agreement with experimental manipulations of barnacles at this site [51]. In light of the good agreement between observed and modeled dynamics (Fig. 5) and because our focus here is on single species dynamics, we did not account for these facilitation effects in detail.

The DLS driving our observed dynamics is not well characterized by most current explanations of population fluctuation: complex non-linear deterministic dynamics or external stochastic forces. We found no evidence for complex deterministic dynamics generating the fluctuations that we observed; instead, the underlying deterministic skeleton predisposed the system to strongly stable dynamics. Although adding a random stochastic component to the dynamics created fluctuations, these fluctuations did not produce the strong semi-predictable pattern observed in the data, but generated a pattern of smaller-amplitude noise around a deterministic stable point. Hence, exploring the nature of DLS both empirically and theoretically might be an interesting avenue of future research. For this to be the case, two questions need to be answered. First, is DLS regularly present in natural populations? Second, does the presence of DLS change the properties of ecological systems in important ways?

We suspect that DLS might have been casually noticed by many investigators interested in characterizing the dynamics of natural populations in the form of poorly behaved (heteroscedastic) residuals observed during model fitting exercises (e.g., Figs. 3, 6A, S1). Indeed, analyses using variance-weighted regression to address heteroscedasticity are not unusual, but their implications for population dynamics are usually not explored. Traditionally, ecologists have focused on characterizing the mean behavior of systems; hence, heteroscedastic residuals are usually treated as a nuisance in the process of generating an adequate description of population dynamics. Our experience with the role that DLS plays in mussel populations suggests that more explicit characterization and consideration of the processes underlying patterns of variability around the mean behavior of ecological dynamics will be a profitable enterprise leading to biological insight into species of interest. In the example we provide here, characterizing the variability allows prediction of more realistic population dynamics (see also [14], [52], [53]), and points to the importance of readily identifiable mechanisms that create this pattern.

We also hypothesize that DLS is a common feature of many natural populations because several disparate general mechanisms that affect population dynamics and lead naturally to such patterns have been described in the literature. The concept of density-vague population change [11] suggests that many species exhibit largely stochastic variation over a broad range of intermediate densities, but that at very high and low densities, deterministic processes dominate the dynamics to permit population persistence. Clearly this hypothesis suggests that stochasticity is a function of density, but of opposite form to the pattern we report here. Competition for refuges from physical stress has also been hypothesized to play a strong role in population dynamics, including the classic work of Andrewartha and Birch [1]. In this scenario, competition at high abundance forces many individuals out of refuges, into areas where they are vulnerable to stochastic fluctuations of the physical environment, but most individuals in the population are sheltered from these forces when abundances are low. Demographic stochasticity [54] is expected to be stronger at low than high population size, potentially generating a density-dependent pattern.

Our own study suggests several other general mechanisms that can lead to DLS. Size structure might create DLS both because the impacts of physical factors can scale with body size [55]–[57], and because changes in body size through development are often associated with ontogenetic niche shifts [58], subjecting different components of the population to different regimes of environmental conditions. Demographic parameters associated with different life stages can have strikingly different variabilities and strengths of density dependence [59]–[63]. Except in special cases (when population growth is constant), size or stage structure will vary with population size. Often size or stage structure will be related to density, as growing populations starting at low density are likely to be dominated by small size classes derived from recent reproduction, whereas more static populations at high abundance are dominated by large adults [64].

Density-linked stochasticity might also be a hallmark of local interactions among organisms, particularly when these involve interactions with the physical environment. The pattern of wave disturbance transmission exhibited in our mussel bed system has parallels in other systems. For example, fire is more likely to spread when trees or shrubs are densely packed together [22], [65], and trees blown down by the wind are likely to knock over neighbors at high density [66]–[69]. Periphyton tends to be sloughed off during floods or strong wave wash at high biomass because of reduced attachment area relative to biomass and increased basal sediment accumulation [70]–[72]. If disease is considered a stochastic factor external to the system, disease transmission is well known to increase with density as contact rate increases [23], [73], [74]. Similarly, aggregative responses of generalist predators would increase the chances of local predation events [21]. Conversely, organisms in some situations might facilitate others at high density by ameliorating physical factors [66], [75], thereby reducing stochastic mortality at high density.

These examples emphasize that the perception of DLS depends on the level of detailed data available to model population dynamics. For example, when detailed data on stage structure or spatial structure are available, models that explicitly include simple density-independent stochasticity for several detailed processes might yield DLS when the population is viewed at an aggregated level [14], [30]. While modeling this complexity is often desirable when adequate data are available, such detailed data are frequently unavailable for populations of interest. Hence, incorporating DLS into population analyses in these situations should provide more realistic dynamics, and both motivate and target more detailed data collection to explore processes generating the dynamics.

Superficially, DLS appears similar to Taylor’s Power Law (TPL [76], [77]) in that density and variability are related. TPL, however, describes the relationship between the overall time series means of different species and their temporal departures from the means, whereas DLS concerns variation in the expected change in a population given a specific starting population size. Although DLS might contribute to TPL, sampling effects from bounded parameters can also cause this pattern and do not constitute DLS. More generally, DLS implies that the parameters describing variability in the system are functions of the dependent variable being modeled (population abundance); it does not refer to distributions (e.g., simple log-normal, Poisson or binomial) which have shapes described by constants, but which are well known to exhibit variance that changes with the mean. In these distributions, shape parameters do not equate to variance.

The broader ecological implications of DLS are less certain and deserve further investigation. Our study shows that this phenomenon is capable of generating strong fluctuations in populations through time, and has statistical implications for how models are parameterized from dynamic population data (see also [10]). How its effects translate to the broader system are less certain, and will depend on whether the species affected by the phenomenon impact other components of the ecosystem in which they exist. If the affected species interact strongly, then they might serve as key ecosystem components that amplify or dampen the effects of environmental stochasticity [27], [78], [79]. Also, linking stochastic elements at specific densities might be extremely effective at promoting spatial asynchrony among populations. Asynchrony is likely to facilitate coexistence and species persistence at broader scales through metapopulation and metacommunity mechanisms [24], [80], [81]. Aside from the spatial aspect of the effect, if stochasticity is higher at high densities, as in our example, it might also facilitate coexistence through temporal storage effect mechanisms [80], [82] by introducing high variability to the system at a point where competitive displacement is most likely. We suspect that other ecologically important effects will be uncovered as the phenomenon is studied in more detail. Regardless of how the phenomenon of DLS manifests in ecosystems, our results contribute to the emerging realization in ecology that in order to understand many dynamic systems, changes in variability must be modeled in tandem with the associated mean process [83]. Focusing on the development of models for these two components will allow researchers to more flexibly test and expand their mechanistic understanding of ecological dynamics.

## Supporting Information

Figure S1 **Comparison of fits of different functions to first-order data describing the proportional area covered by mussels in a plot at time t as a function of the proportional area covered the previous year, assuming constant variance.** Blue curve: non-parametric LOESS fit, Green curve: modified Ricker model with linear density-dependence and outside immigration (Equation 2), Orange curve: modified Hassell model with immigration and additional non-linear term (Equation 3).
(TIF)

Figure S2 **Examples of probability mass functions of beta-binomial distributions with different control parameter values (a, b) for a sample range of 100.**
(TIF)

Figure S3 **Change in shape of the beta-binomial distribution with fixed variance (σ^2^ = 100) as the mean of the distribution changes.** Note the change in scales for the different graphs.
(TIF)

Figure S4 **Residual deviation of A) observed population size from mean (uniform stochastic) model predictions and B) abundance predicted from a spatially explicit model of the mussel bed** [**30**] **compared to a mean field Markov chain model lacking explicit local interactions** [**31**] **.** Data in B) are abundances from 16 randomly placed quadrats equivalent to those used to collect empirical data, taken over 17 time steps (years). Note the expansion of variance around the relationship at mussel cover >0.8 in the empirically observed pattern (A) and when spatially localized interactions are modeled (B).
(TIF)

Table S1 **Comparisons of best fitting (first-order, non-linear, density-linked stochastic) models for first- or fourth-order data, and parameter estimates derived from model averaging.**
(DOCX)

Text S1 **The beta-binomial distribution.**
(DOC)

Text S2 **Effects of localized disturbance interactions.**
(DOCX)
