# Supplementary figures and images for: Complex Population Dynamics in Mussels Arising from Density-Linked Stochasticity
Source: PLoS One. 2013 Sep 23;8(9):e75700. doi: 10.1371/journal.pone.0075700 (PMC3781081; doi:10.1371/journal.pone.0075700)

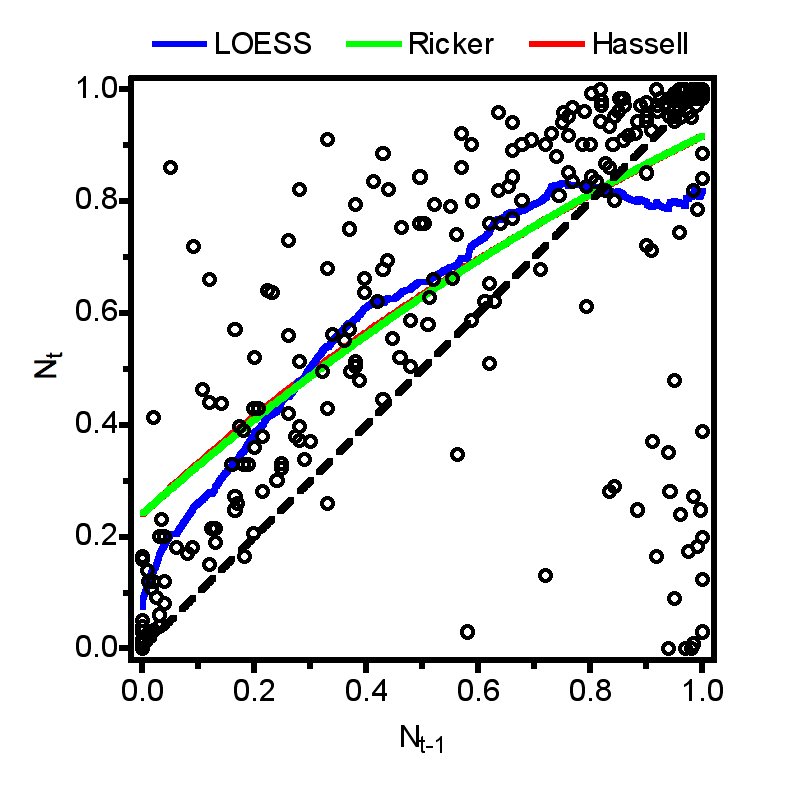

Supplement: Figure S1 — Comparison of fits of different functions to first-order data describing the proportional area covered by mussels in a plot at time t as a function of the proportional area covered the previous year, assuming constant variance. Blue curve: non-parametric LOESS fit, Green curve: modified Ricker model with linear density-dependence and outside immigration (Equation 2), Orange curve: modified Hassell model with immigration and additional non-linear term (Equation 3). (TIF) [file pone.0075700.s001.tif]

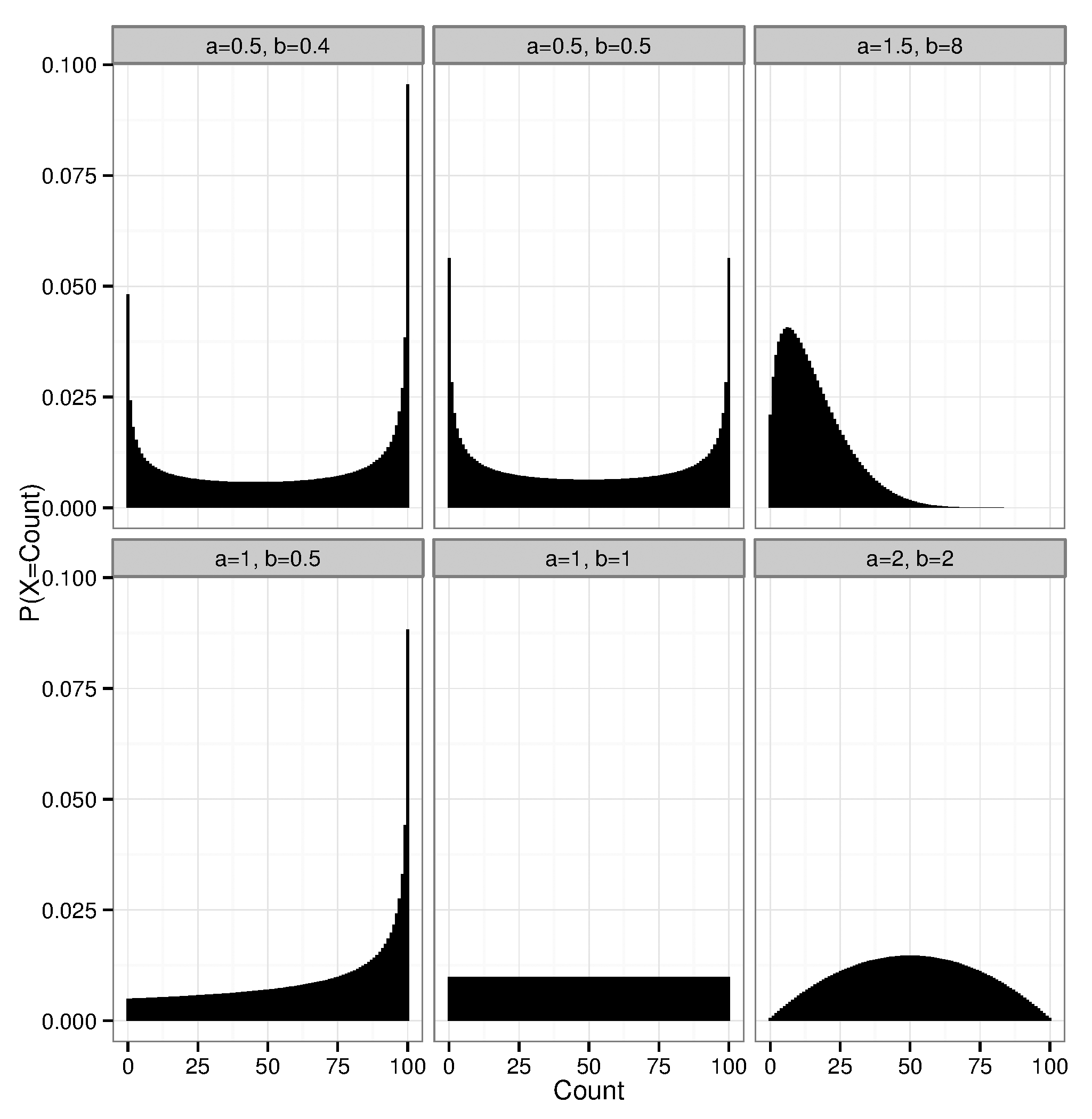

Supplement: Figure S2 — Examples of probability mass functions of beta-binomial distributions with different control parameter values (a, b) for a sample range of 100. (TIF) [file pone.0075700.s002.tif]

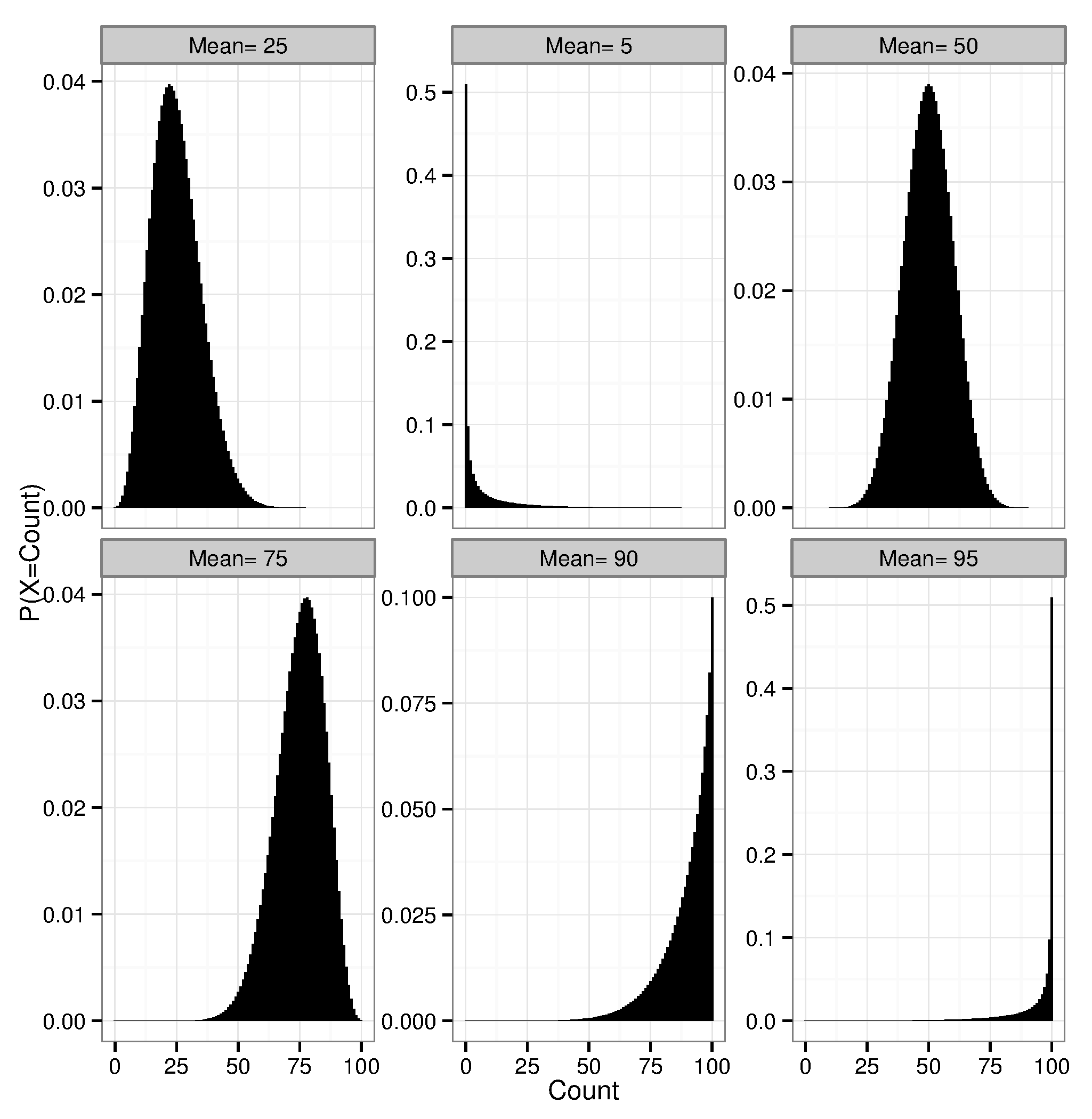

Supplement: Figure S3 — Change in shape of the beta-binomial distribution with fixed variance (σ2 = 100) as the mean of the distribution changes. Note the change in scales for the different graphs. (TIF) [file pone.0075700.s003.tif]

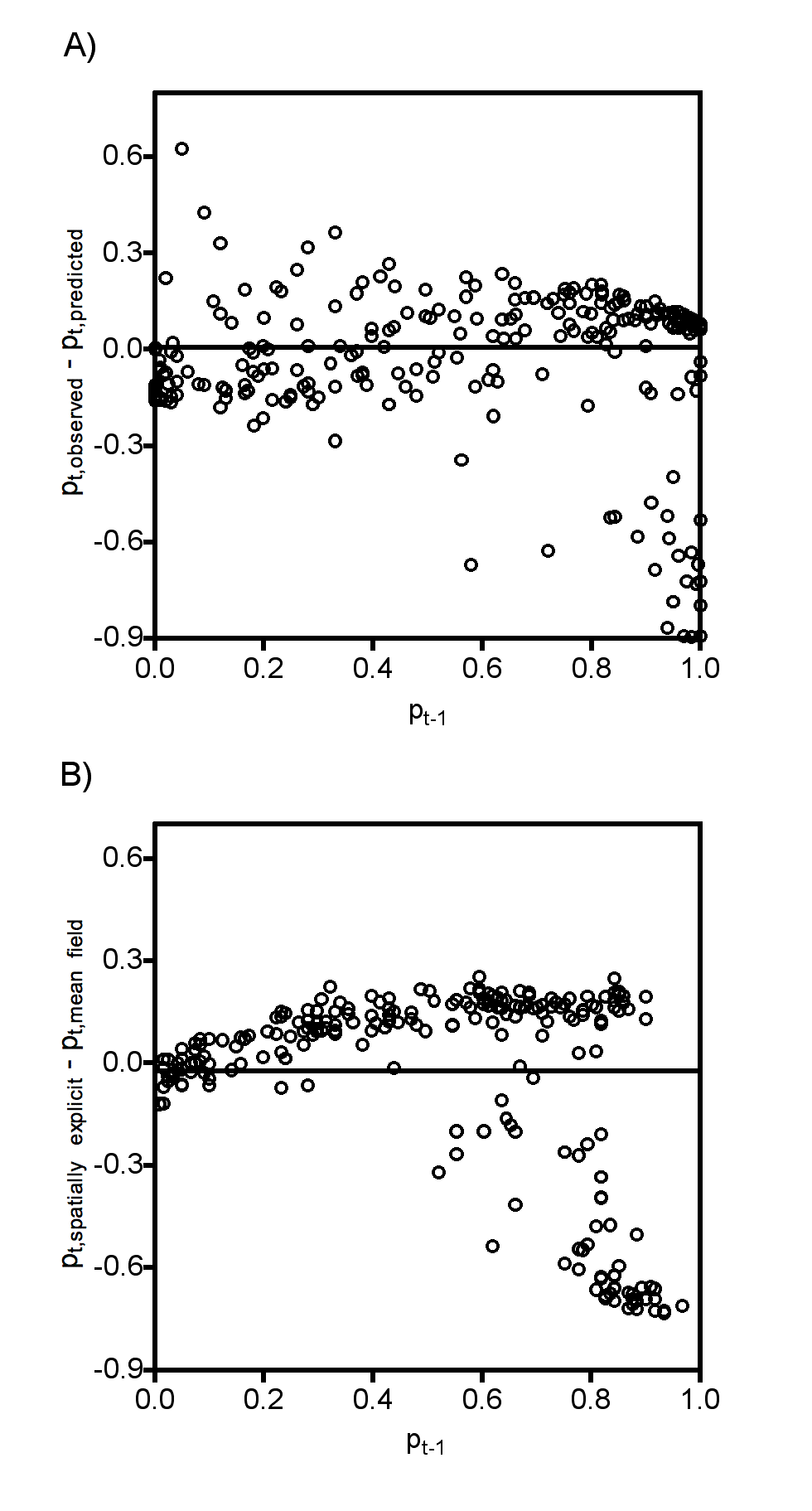

Supplement: Figure S4 — Residual deviation of A) observed population size from mean (uniform stochastic) model predictions and B) abundance predicted from a spatially explicit model of the mussel bed [30] compared to a mean field Markov chain model lacking explicit local interactions [31] . Data in B) are abundances from 16 randomly placed quadrats equivalent to those used to collect empirical data, taken over 17 time steps (years). Note the expansion of variance around the relationship at mussel cover >0.8 in the empirically observed pattern (A) and when spatially localized interactions are modeled (B). (TIF) [file pone.0075700.s004.tif]
