# Supplementary material for: Complex Population Dynamics in Mussels Arising from Density-Linked Stochasticity
Source: PLoS One. 2013 Sep 23;8(9):e75700. doi: 10.1371/journal.pone.0075700 (PMC3781081; doi:10.1371/journal.pone.0075700)
Supplement: Table S1 — Comparisons of best fitting (first-order, non-linear, density-linked stochastic) models for first- or fourth-order data, and parameter estimates derived from model averaging. (DOCX) [file pone.0075700.s005.docx]

Table S1. Comparisons of best fitting (first-order, non-linear, density-linked stochastic) models for first- or fourth-order data, and parameter estimates derived from model averaging.

|  | 4 year lag data | | | |  |  | 1 year lag data | | | | | | | | |  |
| --- | --- | --- | --- | --- | --- | --- | --- | --- | --- | --- | --- | --- | --- | --- | --- | --- |
|  | |  | Model Averaged | | |  | |  | | Model Averaged | | | |  |  |  |
|  | | Best Fit | Mean | CI | |  | | | Best Fit | | Mean | | CI | |  |  |
| I | | 0.053 | 0.052 | 0.047, 0.057 | |  | | | 0.071 | | 0.069 | 0.063, 0.076 | | | | |
| r | | 0.522 | 0.530 | 0.508, 0.552 | |  | | | 0.600 | | 0.599 | 0.592, 0.605 | | | | |
| α_1_ | | -0.524 | -0.533 | -0.558, -0.508 | |  | | | -0.615 | | -0.611 | -0.625, -0.597 | | | | |
| β | | 0* | 0.002 | -0.0005, 0.004 | |  | | | 0.014 | | 0.011 | 0.006, 0.015 | | | | |
| c | | 0* | 44.84 | -69.42, 159.10 | |  | | | 28.62 | | 22.14 | 10.52, 33.77 | | | | |
| α_2_ | | 0* | -2x10^-5^ | -7x10^-5^, 2x10^-5^ | |  | | | 0* | | 0* | 0* | | | | |
| β_2_ | | 0* | 0.001 | -0.001, 0.003 | |  | | | 0* | | 0* | 0* | | | | |
| c_2_ | | 1* | 1.011 | 0.968, 1.053 | |  | | | 1* | | 1* | 1* | | | | |
| α_3_ | | 0* | -1x10^-5^ | -4x10^-5^, 1x10^-5^ | |  | | | 0* | | 0* | 0* | | | | |
| β_3_ | | 0* | 0.002 | -0.002, 0.006 | |  | | | 0* | | 0* | 0* | | | | |
| c_3_ | | 1* | 0.998 | 0.990, 1.006 | |  | | | 1* | | 1* | 1* | | | | |
| α_4_ | | 0* | 1x10^-5^ | -1x10^-5^, 4x10^-5^ | |  | | | 0* | | 0* | 0* | | | | |
| β_4_ | | 0* | 4x10^-7^ | -9x10^-7^, 2x10^-6^ | |  | | | 0* | | 0* | 0* | | | | |
| c_4_ | | 1* | 1.004 | 0.987, 1.022 | |  | | | 1* | | 1* | 1* | | | | |
| *x* | | -2.959 | -2.962 | -3.165, -2.760 | |  | | | -2.570 | | -2.596 | -2.756, -2.435 | | | | |
| *y* | | -2.023 | -1.991 | -2.200, -1.781 | |  | | | -2.308 | | -2.288 | -2.516, -2.060 | | | | |
| *z* | | 13.13 | 13.32 | 11.957, 14.683 | |  | | | 8.983 | | 8.859 | 7.992, 9.726 | | | | |
| *μ_d_* | | 0.393 | 0.393 | 0.372, 0.415 | |  | | | 0.392 | | 0.391 | 0.370, 0.413 | | | | |
| *σ^2^_d_* | | 0.090 | 0.090 | 0.088, 0.092 | |  | | | 0.090 | | 0.090 | 0.088, 0.092 | | | | |
| *j* | | -3.143 | -3.133 | -3.434, -2.832 | |  | | | -3.172 | | -3.115 | -3.473, -2.756 | | | | |
| *k* | | 2.225 | 2.213 | 2.000, 2.427 | |  | | | 2.288 | | 2.232 | 1.930, 2.533 | | | | |

*parameter fixed a priori.
