# Supplementary material for: Complex Population Dynamics in Mussels Arising from Density-Linked Stochasticity
Source: PLoS One. 2013 Sep 23;8(9):e75700. doi: 10.1371/journal.pone.0075700 (PMC3781081; doi:10.1371/journal.pone.0075700)
Supplement: Text S1 — The beta-binomial distribution. (DOC) [file pone.0075700.s006.doc]

Supporting Text S1.

*The Beta-Binomial Distribution*

Beta-binomial distributions provide a flexible framework for modeling bounded discrete distributions (Crowder 1978, Kendall 1998). When bounded by the interval [0,1], the beta-binomial can be thought of as a description of a distribution of events where the outcome of an individual event has two possibilities (present/absent, alive/dead, etc.), the binomial portion of the distribution, but that the probability of the event occurring varies, for example as the environment fluctuates, which is described by the beta portion of the distribution (Richards 2008). The benefit of using the beta-binomial over a continuous beta distribution in situations such as ours is that observations of 0 and 1 are permitted. The equations and discussion that follow are only strictly applicable to a distribution bounded by [0,1].

The beta-binomial distribution is controlled by two positive-valued shape parameters (*a, b*), which define the beta distribution component, and the sample size (*N*), which defines the subsequent binomial component of the distribution. Depending on the shape parameters, the resulting distribution can be approximately uniform (*a* = *b* = 1), symmetrically unimodal (*a* = *b* > 1), bimodal (*a*, *b* < 1) or skewed (*a* ≠ *b*) (e.g. Fig. S2). For convenience and efficiency of model fitting, it is sometimes useful to work with the sum of the shape parameters (*φ* = *a* + *b*) (Morris 1997).

The shape parameters of the beta portion of the distribution are related to the mean (*μ*) and variance (*σ^2^*) of the distribution. Hence, when *σ^2^* is fixed the shape of the distribution varies from strongly skewed right to unimodal to strongly skewed left as *μ* increases from its minimum to maximum value (0-1 in our analyses; Fig. S3).

The mean and variance of the beta portion of the distribution are related to the shape parameters through the following relationships:

Equations S1

or, after rearranging:

Equations S2.

Hence, we can define the beta-binomial distribution given an expected value (e.g., a function describing predicted mean population size) and rules for the behavior of the variance of that function. We apply these relationships in our model fitting procedure by defining a population dynamic function to fit and rules for how variance behaves across that function, following either traditional assumptions (*σ^2^* is a constant) or assumptions that variation depends on density.

Under constant *σ^2^*, the shape parameters vary with the mean such that:

(0 < *μ* < 1) Equation S3.

Note that this equation accounts for constant process error (beta function portion of the distribution), not the sampling error (binomial portion), which changes the overall distribution such that variance is slightly higher at intermediate (≈0.5) values of *μ* compared to values near the ends of the distribution (0 or 1).

When *a* and *b* are constants, *σ^2^* naturally varies with mean predicted abundance, such that variance approaches 0 when *μ* approaches 0 or 1. This pattern qualitatively does not describe our data, and model fitting using this special case of varying *σ^2^* consequently provides very poor fits. For this reason, we do not discuss this situation further.

For more flexible density-linked variance patterns, we used the relationship:

Equation S4.

Focusing on *φ* in this equation, rather than *σ^2^* directly, still allows variance to increase or decrease with abundance (*p_t-1_*), but also provides a smooth function that increases the efficiency of fitting algorithms, and that helps maintain constraints that the shape parameters be positive and that the variance distribution be unimodal and positive during the fitting procedure. The negative sign in the exponential term is included so that increasing any of variance control parameters (*x, y, z*) results in increasing variance, thereby making interpretation of parameters easier.

The parameters that maximize the fit of a model given a set of data should be those that maximize the probability mass function (PMF) for the beta-binomial, or alternatively minimize, the –log(PMF). For our 16 quadrats with *t_max_* time intervals (15-17), the latter is given by the equation:

Equation S4,

where *Ω_q,t_* is the observed abundance in quadrat *q* at time *t*, *N* is the sample size in quadrat *q*, *Beta* is the beta function with shape parameters *a*, and *b*. Note that because *a* and *b* depend on the expected function and variance, which change with differing density in each quadrat at each sample point, these values vary for each different sample point in each different quadrat.

The combinatorial term in Equation S4 creates computational accuracy problems due to very large factorial numbers at high N. Therefore in our calculations, we used a form with a large number approximation to estimate the PMF:

Equation S5.

Beta-Binomial References

Crowder MJ (1978) Beta-binomial ANOVA for proportions. Appl Stat-J Roy St C 27: 34-37.

Kendall B (1998) Estimating the magnitude of environmental stochasticity in survivorship data. Ecol Appl 8: 184-193.

Morris WF (1997). Disentangling effects of induced plant defenses and food quantity on herbivores by fitting nonlinear models. Am Nat 150: 299–327.

Richards S (2008) Dealing with overdispersed count data in applied ecology. J Appl Ecol 45: 218-227.
