# Supplementary material for: Complex Population Dynamics in Mussels Arising from Density-Linked Stochasticity
Source: PLoS One. 2013 Sep 23;8(9):e75700. doi: 10.1371/journal.pone.0075700 (PMC3781081; doi:10.1371/journal.pone.0075700)
Supplement: Text S2 — Effects of localized disturbance interactions. (DOCX) [file pone.0075700.s007.docx]

Supporting Text 2

*Effect of Localized Disturbance Interactions*

Prior studies (Robles et al. 1995, Wootton 2001b) suggest one mechanism that might contribute to DLS in mussel beds: the transmission of wave disturbance among adjacent individuals when neighbors attach their byssal threads to each other rather than to the rock substrate. Wootton (2001b) developed, parameterized and analyzed a cellular automata model of this mussel bed system to explore the role of spatially localized interactions within the community. This model, which simulated the community dynamics within 6 x 6 cm cells of an intertidal bench ~6 x 18 m, effectively recaptured spatial patterning of the system when wave disturbance was propagated by local interactions among mussels via mutual byssal thread attachment. If spatially localized interactions underlie key aspects of the observed DLS, then when spatial points in the simulation are aggregated into population data at the scale of the quadrats used in this study, DLS should emerge.

To convert the cellular automata to a population model we randomly placed 16 10 x 10 cell quadrats on the 100 x 300 grid used in the model to scale the system to a typical rock bench at this site. We then ran the cellular automata for 500 simulated years to eliminate transient patterns and subsequently collected data on predicted mussel cover in each quadrat for a period of 17 simulated years. To isolate the effects of local interactions on population dynamics, we compared these dynamics to a mean-field model lacking spatially local interactions (i.e., a spatial version of a Markov chain model parameterized for the system using the same initial conditions; Wootton 2001a, 2004, 2013).

Essential features of observed DLS emerged when spatially-localized interactions act in concert with stochastic disturbance events (Fig. S4). This pattern indicates that spatially-local interactions are the cause of the pattern in the simulations and can contribute key aspects of the observed dynamics.

Supporting Text 2 References

Robles C, Sherwood-Stephens R, Alvarado M (1995) Responses of a key intertidal predator to varying recruitment of its prey. Ecology 76: 565-579.

Wootton JT (2001a) Local interactions predict large-scale pattern in an empirically-derived cellular automata. Nature 413: 841-843.

Wootton JT (2001b) Prediction in complex communities: analysis of empirically-derived Markov models. Ecology 82: 580-598.

Wootton JT (2004) Markov chain models predict the consequences of experimental extinctions. Ecol Lett 7: 653-660.

Wootton JT (2013) An experimental test of multi-species Markov models: Are barnacles long-term facilitators of mussel bed recovery? Bull Mar Sci 89: 337-346.
